# Evidence-based selection process to the Master of Public Health program at Medical University

**DOI:** 10.1186/s12909-017-1007-z

**Published:** 2017-09-11

**Authors:** Mariusz Panczyk, Grzegorz Juszczyk, Aleksander Zarzeka, Łukasz Samoliński, Jarosława Belowska, Ilona Cieślak, Joanna Gotlib

**Affiliations:** 1 Division of Teaching and Outcomes of Education, Faculty of Health Science, Medical University of Warsaw, Zwirki i Wigury 61, 02-091 Warsaw, Poland; 2 Department of Public Health, Faculty of Health Sciences, Medical University of Warsaw, Warsaw, Poland

**Keywords:** School admission criteria, Public health professional, Educational status

## Abstract

**Background:**

Evaluation of the predictive validity of selected sociodemographic factors and admission criteria for Master’s studies in Public Health at the Faculty of Health Sciences, Medical University of Warsaw (MUW).

**Methods:**

For the evaluation purposes recruitment data and learning results of students enrolled between 2008 and 2012 were used (*N* = 605, average age 22.9 ± 3.01). The predictive analysis was performed using the multiple linear regression method. In the proposed regression model 12 predictors were selected, including: sex, age, professional degree (BA), the Bachelor’s studies grade point average (GPA), total score of the preliminary examination broken down into five thematic areas. Depending on the tested model, one of two dependent variables was used: first-year GPA or cumulative GPA in the Master program.

**Results:**

The regression model based on the result variable of Master’s GPA program was better matched to data in comparison to the model based on the first year GPA (adjusted R^2^ 0.413 versus 0.476 respectively). The Bachelor’s studies GPA and each of the five subtests comprising the test entrance exam were significant predictors of success achieved by a student both after the first year and at the end of the course of studies.

**Conclusions:**

Criteria of admissions with total score of MCQs exam and Bachelor’s studies GPA can be successfully used for selection of the candidates for Master’s degree studies in Public Health. The high predictive validity of the recruitment system confirms the validity of the adopted admission policy at MUW.

**Electronic supplementary material:**

The online version of this article (10.1186/s12909-017-1007-z) contains supplementary material, which is available to authorized users.

## Background

An adequate competence level of public health specialists is the key element to ensure scientific advancement and effective introduction of a well-planned health policy [1, 2]. In addition to a well-designed Public Health program taught by qualified university staff, an accurate and reliable candidate selection procedure is indispensable for effective education. If these conditions are fulfilled, students are more likely to acquire competences required to act as experts, advisors or consultants for public health institutions and to perform executive functions in such organizations. In this context, all schools and programs have admission criteria to select the best candidates for their Public Health programs. However, we do not have enough evidence to support that each requisite or criterion specific to the programs is a predictor of academic success [3]. Moreover, although some of the criteria are objective, much subjectivity is present in the admission process.

The issue of an appropriate adjustment of the admission policy to the educational requirements of medicine-related programs has been the subject-matter of scientific analyses for several decades [4, 5]. Practice shows, however, that it is still a very important problem that has not been resolved completely [6]. We could cite here a statement that is found in one of the papers of Judy Searle and Jane McHarg: “Just pick the right students and the rest is easy!” [7]. A considerable number of analytical and critical studies have been conducted on these issues, in particular as regards the selection of appropriate candidates for medical studies, and to a lesser degree regarding such programs as dentistry [8] and pharmacy [9]. There are a number of available studies on good recruitment practices for nursery and obstetrics as regards health professions [10]. In the case of selection of appropriate candidates for Public Health studies there are no reliable and long-terms analyses of recruitment strategies [3]. Appropriate selection tools for Public Health studies should be developed based on solutions and good practices described in papers on other health professions.

With an appropriate set of selection criteria for a given study program it is possible to implement an admission policy to a university that is relevant to the university’s objectives. The choice of such criteria is directly connected with the assessment of validity which is to be understood as the degree of conformity with which an educational measurement tool measures what it was designed to measure. Therefore, validity assessment is the usefulness of a given criterion in the assessment of a specific set of features and characteristics of an exam taker [11]. There is no precise method for measuring validity, and instead only a certain indirect assessment is available. In this paper, the analysis of the validity of the MCQs entrance test was based on the examination of predictive validity. Predictive validity is a type of criterion validity that addresses how a test like MCQs exam predicts later criteria, such as GPA or licensing-exam scores.

Admission criteria must, on the one hand, ensure uniform conditions and a fair assessment of each candidate, while, on the other, they must reliably and validly measure features that are important for successful undertaking of studies [12]. From among a diversified array of various criteria, to select candidates most universities use a grade point average (GPA) and standardized aptitude tests (e.g. multiple-choice questions exam), mini-interviews and written essays [13]. In selection of the best candidates, the best criteria are those that offer considerable specificity helping to prevent persons with an inadequate level of entry competences from beginning studies [3]. The strive to create an effective method of selecting the best candidates who may be, with a high degree of probability, expected to be professionally successful in the future is of particular importance in education of healthcare professionals [3, 6, 7].

One of the most important elements in assessing the quality of the admission process is the estimation of the predictive validity that is usually based on correlation analyses and regression models [14–16]. The methodology of this estimation allows one to verify hypotheses about the extent to which the measurement of entry competences in the recruitment process can accurately predict certain quantitative criteria in the future (e.g. GPA from the course of studies). The selection of an appropriate final criterion is connected with a necessity to identify a certain objective measure of a “student’s success”. For this reason, the criterion variable is very often a combination of different measurements, as is the case with the grade point average that reflects an average score of a student’s achievements in a given period of education.

Among the available literature data (PubMed, Scopus, Web of Science, searched in May 2015, key words: “*Education, Graduate*” AND “*School Admission Criteria*”) concerning studies of the quality of recruitment systems for university studies connected with education of health science specialists, only some papers relate strictly to the Public Health program [3, 17, 18]. This means that there is limited evidence corroborating the validity of the applied admission policy for Public Health studies. Therefore, in line with a recommendation of the National Association for College Admission Counselling, educational establishments should examine validity of admission tests and communicate their findings to other universities [19]. In light of this recommendation, it is striking that the field of public health, which tries to base its progress on rigorous scientific studies, does not apply this same rigorous approach to the selection of its students [3].

A good admission policy should, like evidence-based medicine, rely on credible empirical data. In accordance with the concept of *Evidence-Based Admission Criteria* proposed by Lamadrid-Figueroa et al., the admission policy of a university should be created on the basis of ongoing evaluation of the criteria used for selecting candidates for a particular program of studies [3]. The gravity of the problem connected with poor assessment of candidates’ entry competences is illustrated by the scale of financial losses incurred due to the high attrition rate among students of nursery and obstetrics in Scotland [20]. Unfortunately, only limited data on validation of admission criteria for candidates for Public Health are available. The Sabin & Scottish Recruitment Retention Delivery Group lists various reasons contributing to student attrition such as gender, age, educational qualifications, personality and “stage of programme”. Further, a number of key areas have been highlighted as supporting student retention including admission and selection processes [10]. This is why the evaluation of the efficacy, reliability and validity of admission tests as selection tools for recruitment of students is so important.

A number of long-term benefits can be derived from studies of attrition and predictive factors of university success. An analysis of attrition and identification of students with both personal and academic problems could help with evidence-based selection processes and launching support services at medical universities [6].

### Aim of study

Results of 5-year retrospective studies presented in this paper concern the predictive validity of selected sociodemographic factors and admission criteria for the Master’s studies as part the Public Health program at the Faculty of Health Sciences, the Medical University of Warsaw.

## Methods

### Description of the admission selection process

The Faculty of Health Sciences of the Medical University of Warsaw (MUW) is one of 11 academic units in Polish medical universities that educate Public Health professionals in first and second degree studies [21]. Annually, over 1 hundred MUW graduates earn the Master’s degree in Public Health in three specializations: *General*, *Health Promotion and Epidemiology*, and *Management in Health Care*.

Candidates admitted to the qualification process for the Master’s degree studies may include bachelor graduates of Public Health, medical programs and other study programs meeting the curriculum minimum of the Public Health program. In practice, graduates of such programs as Dietetics and Food Science, Electroradiology, Emergency Medicine, Nursing and Midwifery, Physiotherapy, and Dental Hygiene and Dental Technology are admitted to qualify for second-degree studies.

Between 2008 and 2012, the MUW recruited candidates on the basis of results of a multiple-choice questions (MCQs) exam and the Bachelor’s studies GPA (and in the course of recruitment between 2008 and 2011 the result of the Bachelor’s examination was additionally taken into account). Each MCQs exam consisted of 50 questions prepared in the format of the best answer from a list of possible answers and each question had five multiple-choice options. In each of the MCQs exams, in accordance with test content outlines, there were five subtests. The examination questions were categorized into two domains: base knowledge and subject-area knowledge and the ability to apply information in practice.

In the analysis of the curriculum objectives for the master’s studies according to the criteria proposed by the ASPHER (*Association of Schools of Public Health in the European Region*), certain significant areas of expertise and skills were identified that are necessary for undertaking Public Health studies successfully. It seems that the isolated theme areas: (1) *Epidemiology,* (2) *Organization in Healthcare,* (3) *Methodology and Statistics,* (4) *Health and Human Nutrition,* and (5) *Social Science* are a good prerequisite for successful studies.

### Description of the master in public health program curriculum

Public health is an interdisciplinary study program that includes elements of health studies, social science, medical studies, and physical education studies. The list of competences that a program graduate should have was published by the ASPHER in 2008. It includes a list of expected 334 practical and 246 theoretical competences, grouped into six main areas [2, 22].

The Master’s studies in Public Health, compared to the Bachelor’s studies, considerably extend the scope of knowledge of and skills in this area. The program includes advanced courses in economics, management, epidemiology, biostatistics, research methodology and health promotion. It also comprises substantial elements of public health practice. Master graduates also have the knowledge of health care law, evidence based health policy, financing and management in health care. In the course of studies, students are also taught how to appropriately and effectively identify social and environmental health factors, as well as interdependencies between human health, environmental conditions, the welfare system and the socioeconomic situation of the State.

Subjects taught in the course of the Master’s studies are offered as lectures, seminars and practical classes. With some subjects, such as health sociology or social policy, lectures are predominant. With other courses, such as biostatistics or research methodology, practical classes are held in small groups of about ten. During the latter courses, students also carry out their own research projects and analyse them statistically.

### Data collection

Recruitment data and education results of students who started their Master’s studies in Public Health between 2008 and 2012 (*N* = 605, average age 22.9 ± 3.01) were analysed. The vast majority of the study group were women (86.8%) and graduates who completed their Bachelor’s studies outside the MUW (73.6%). Nearly two-thirds of the students had a bachelor’s degree in Public Health. Detailed characteristics of the group under study are presented in Table 1.

**Table 1:** Characteristics of the group of students who started the Master’s studies in Public Health

	2008	2009	2010	2011	2012	TOTAL
N	104	134	124	110	133	605
Women	87	114	104	102	118	525
Men	17	20	20	8	15	80
Mean age on entry (SD)	22.4 (0.89)	22.9 (2.98)	23.2 (2.97)	23.0 (3.83)	23.0 (3.35)	22.9 (3.01)
Bachelor Degree						
Medical University of Warsaw	18	30	28	34	50	160
Other	86	104	96	76	83	445
Modes of study						
Full-time studies	104	101	85	82	114	486
Part-time studies	0	33	39	28	19	119
Professional title						
Public Health	64	89	85	69	83	390
Other	40	45	39	41	50	215

*SD* Standard deviation

Data concerning such variables as gender, age, the location where the bachelor’s studies were completed or the professional degree held were all obtained from application forms filled in by candidates. As regards education results, data concerning the GPAs achieved by students both after the first year and during the entire period of studies were collected. The above data were recorded in the Central Student Database used for supporting administrative services for students and course of studies.

### Predicting academic success

Prediction or forecasting is, according to William Wiersma, “*estimation of scores on one variable from information about one or more other variables”* [23]. Two types of variables are used in this kind of analyses: dependent variable and independent variable(s) (predictor(s)) [24]. In the methodology of psychometric research, a dependent variable is this kind of feature we can conclude about on the basis of values accepted by a predictor (or predictors) [24].

Student’s GPA for the first year is most often used in predictive studies on the efficiency of the admission procedure as a dependent variable that accounts for academic success [25–29]. Student’s education outcomes in the first two semesters are considered to be an indicator to what extent a student met academic requirements imposed on students of a particular major. GPA for all examinations set out in the curriculum (cumulative GPA) constitutes another variable allowing for a relatively objective assessment of academic success of a student. This variable is a frequently used outcome variable in studies concerning validation of admission criteria [3, 30, 31].

With reference to the factors determining academic success, there are various groups of predictors that directly or indirectly influence future educational progress of a student. In accordance with the NURS (Nursing Universal Retention and Success) model developed by Marianne Jeffreys to assess students and graduates in Nursing, [32] the following groups of independent variables can be distinguished: background variables (e.g. gender, age, professional degree held (Bachelor’s degree)), environmental variables (e.g. economic variables, work while studying, family responsibilities), academic variables (e.g. educational potential: the Bachelor’s studies GPA, the score result of the entry examination, study mode), internal variables (e.g. academic self-efficacy, goals, attitude). As seen above, the NURS model is based on a number of variables for which clear indications concerning their impact on the success/failure in education are available [32].

Two predictive models (model I and II) proposed in the present paper were based on four predictors from the sociodemographic group (background variables) and eight predictors from the group of variables associated with the admission process (academic variables). The group of background variables: (1) gender, (2) age, (3) professional degree held (Bachelor’s degree), and (4) the school in which Bachelor’s studies were completed. Eight academic variables connected with the admission process were used: (1) the Bachelor’s studies GPA, (2–6) the score result of the entry examination broken down by five theme areas, (7) the recruitment year and (8) the preferred study mode (full-time/part-time). The Bachelor’s studies GPA was calculated on the basis of data in a *Diploma Supplement*, a document presenting an overview of a studies program and a candidate’s achievements. Owing to different methodologies used by universities to calculate the GPA as either an arithmetic mean or weighted average, the GPA calculated as an arithmetic mean of grades awarded in examinations in subjects provided for in a university’s studies program was used an admission criterion. The Bachelor’s studies GPA did not take account of grades awarded for a Bachelor’s thesis or a Bachelor’s examination.

Depending on the tested model, one of the two outcome variables were used: (1) the GPA value obtained after the first year of studies (model I) or (2) the cumulative GPA in the Master program (model II). Outcome variables were calculated as weighted averages of grades awarded in final examinations with weights being the number of ECTS (European Credit Transfer and Accumulation System) points awarded to students for completing each subject. According to the Public Health program, the first year of studies comprised examinations in six subjects (29 ECTS) while the second year included examinations in eight subjects (22 ECTS). As confirmed by Panczyk et al. [33] the grading system for examinations subjects in the Master of Public Health program at the MUW has a good internal consistency. The analysis showed an average level of assessment reliability for all subjects – coefficient α = 0.74 with the level of reliability in consecutive years oscillating between 0.67 and 0.81 [33]. So far no analysis of validity of the grading system in Public Health programs has been carried out.

### Statistical analysis

Data for the results in MCQs exams and subtests and Bachelor’s studies GPA were presented using parameters of descriptive statistics (mean, standard deviation, variation coefficient and skewness). In order to determine reliability of MCQs exams, the values of Cronbach’s α and Guttman’s L4 were estimated and the standard error of measurement (SEM) was calculated. Pearson product-moment correlation (r_p_) was used to assess the correlation between the Bachelor’s studies GPA and results in MCQs exams. The predictive analysis was carried out using the multiple linear regression method. The regression model was fitted to the empirical data by the Ordinary Least Squares (OLS) method. As part of the testing of the assumptions for the multiple linear regression method, the degree of the predictor correlation was assessed in the multicollinearity test (Variance Inflation Factor (VIF)) assuming the maximum value of 10 [34]. In addition, an analysis of residuals was performed to test: homoscedascity (White test) [35], normality distribution (Jarque-Bera test) [36] and the degree of the correlation of residuals (Ljung-Box test) [37]. In addition, as part of model diagnostics the occurrence of outliers was assessed by determining the Mahalanobis and Cook’s distance [38, 39]. To interpret the distance, the rules proposed by Larose [40] and Field [34] were used.

All predictors were introduced to the model at the same time. Dichotomous qualitative variables were coded in the binary system. The model statistics were calculated for each predictor. The vector and intensity of significant correlations were interpreted by determining β standardized regression coefficients. The values of adjusted statistics R^2^ and Akaike Information Criterion (AIC) were determined to assess the degree of explanation of variations for both regression models. *P*-values <0.05 were considered statistically significant. All of the statistical analyses were performed using STATISTICA 12.5 (StatSoft©, Inc.) under the MUW licence.

## Results

### Descriptive statistics for independent and dependent variables

Candidates admitted to the Master program obtained various MCQs exam scores, with the mean being always in excess of 25 points. Candidates with higher scores dominated. Distribution of results was typical of a group of persons selected using the criterion of the pass/fail cut-off point (Table 2).

**Table 2:** Descriptive statistics for MCQs exams

	2008	2009	2010	2011	2012
Mean (95% CI)	25.7 (24.7–26.8)	28.3 (27.5–29.1)	27.6 (26.6–28.5)	27.2 (26.2–28.2)	26.4 (25.4–27.4)
SD	5.36	4.72	5.21	5.29	5.79
CV [%]	20.8	16.7	18.9	19.5	21.9
Skewness	−0.568	−0.754	−0.224	0.283	−0.532

*CI* Confidence interval, *SD* Standard deviation, *CV* Coefficient of variation

The level of reliability of MCQs exams as measured by Cronbach’s α oscillated between 0.530 and 0.747, with the highest recorded for 2012. The standard error of measurement amounted to approximately three points. Results of the analysis of internal consistency of measurements using MCQs exams are presented in Table 3.

**Table 3:** Evaluation of the reliability of MCQs exams

	2008	2009	2010	2011	2012
Cronbach’s α	0.530	0.661	0.664	0.677	0.747
Guttman’s L4	0.501	0.666	0.653	0.645	0.812
SEM	3.4	3.0	3.0	3.1	3.1

*SEM* Standard error of measurement

Assessment of the correlation between the Bachelor’s studies GPA of the candidates and their results in MCQs exams reveals a positive correlation. Correlations were statistically significant both within subtests and for the total score showing the highest r_p_ value (Table 4). These results indicate that the admission criterion of the bachelor’s studies GPA is accurate.

**Table 4:** Correlations between the Bachelor’s studies GPA and the results of the MCQs exams

	r_p_
Score in *Epidemiology* subtest	0.20, *P* < 0.001
Score in *Organization in Health Care* subtest	0.24, *P* < 0.001
Score in *Scientific Method* subtest	0.11, *P* < 0.001
Score in *Health and Human nutrition* subtest	0.16, *P* < 0.001
Score in *Social science* subtest	0.20, *P* < 0.001
Total score	0.31, *P* < 0.001

*r*_*p*_ Pearson product-moment correlation

The score average for students after the first year of studies was lower than the score average at the end of the studies (3.63 vs. 3.81), but in the case of the first-year GPA the averages were more varied than in the case of the cumulative GPA (SD of 0.46 and 0.37, respectively). Table 5 presents a detailed list of descriptive statistics parameters for the dependent variables.

**Table 5:** Descriptive statistics for the first-year GPA and cumulative GPA

	First-year GPA	Cumulative GPA
Mean (95% CI)	3.63 (3.60–3.67)	3.81 (3.78–3.84)
SD	0.46	0.37
CV [%]	12.6	9.7
Skewness	0.106	−0.013

*CI* Confidence interval, *SD* Standard deviation, *CV* Coefficient of variation

### Predictive validity

The first of the tested regression models was based on estimation of the impact of background and academic variables (predictors) on the first-year GPA (F = 38.27, *P* < 0.001, standard error of estimation = 0.35). The adjusted value R^2^ for this model was 0.413 (AIC = 454.47), which means that the predictors explained approximately 41% of the variable of the first-year GPA.

The value of Bachelor’s studies GPA constituting one of the admission criteria had the greatest influence on the first-year GPA (β_stand._ = 0.367). In addition, it was found that the value of the first-year GPA depended on the number of points a student had obtained at the entrance exam for particular subtests. The *Health and Human Nutrition* subtest was found to be the strongest predictor of success (β_stand._ = 0.114), whereas for the *Social science* subtest this predictive capacity was the lowest (β_stand._ = 0.069). In general, each additional point obtained by a candidate at the entrance exam gave a higher first-year GPA by between 0.013 to 0.026, depending on subtests. In the case of variables not connected with the process of admission for the Master’s studies, the strongest effect was observed for the qualitative variable: the location of completion of the Bachelor’s studies. For the graduates who earned the Bachelor’s title outside the MUW, significantly lower first-year GPA were noted (β_stand._ = −0.241). Also, the candidate’s professional degree awarded upon completion of the Bachelor’s studies had a significant impact on estimation of the first-year GPA. The students who had Bachelor’s degree in Public Health had better results at the end of the first year of their Master’s studies (β_stand._ = −0.138). Whereas, for the demographic variables *age* and *gender,* no significant effect on the results of education during the first year of the studies was found. A detailed list of the results of the regression analysis for the model with the dependent variable the first-year GPA is presented in Table 6.

**Table 6:** Regression model for the dependent variable the first-year GPA

Variable	b	SE	β	95% CI		*P*-value
Intercept	1.726	0.163	–	–		<0.0001
Age on entry (0 = 23 years; 1 = more than 23 years)	0.030	0.045	0.022	−0.044	0.088	0.5092
Gender (0 = Female; 1 = Male)	0.047	0.044	0.035	−0.029	0.098	0.2869
Modes of study (0 = Full-time; 1 = Part-time)	−0.220	0.043	−0.179	−0.248	−0.111	<0.0001
Professional title (0 = Public health; 1 = Other)	−0.132	0.036	−0.138	−0.212	−0.065	0.0003
Bachelor’s Degree (0 = MUW; 1 = Other)	−0.260	0.042	−0.241	−0.317	−0.165	<0.0001
Bachelor’s studies GPA	0.394	0.039	0.367	0.295	0.438	<0.0001
Score in *Epidemiology* subtest	0.018	0.006	0.102	0.032	0.172	0.0042
Score in *Organization in Health Care* subtest	0.021	0.008	0.100	0.027	0.174	0.0077
Score in *Scientific Method* subtest	0.019	0.007	0.091	0.023	0.159	0.0091
Score in *Health and Human nutrition* subtest	0.026	0.008	0.114	0.045	0.183	0.0013
Score in *Social science* subtest	0.013	0.007	0.069	0.000	0.138	0.0492

*MUW* Medical University of Warsaw

The second tested regression model was based on estimation of the impact of the predictors on the cumulative GPA (F = 49.18, *P* < 0.001, standard error of estimation = 0.27). The adjusted value R^2^ for this model at 0.476 (AIC = 136.26) was slightly higher than that for the first model.

The effect of the variable specifying the location where the candidates completed their Bachelor’s studies was slightly stronger (β_stand._ = −0.287). The analysis of the predictors connected with admission for the Master’s studies shows that, as in the case of the previous model, the Bachelor’s studies GPA variable had the strongest effect on the cumulative GPA (β_stand._ = 0.447). A stronger effect was also observed in the case of the candidates’ results obtained in *Social Science* (β_stand._ = 0.102). A detailed list of the results of the regression analysis for the model with the dependent variable the cumulative GPA is presented in Table 7.

**Table 7:** The regression model for the dependent variable the cumulative GPA

Variable	b	SE	β	95% CI		*P*-value
Intercept	1.912	0.124				<0.0001
Age on entry (0 = 23 years; 1 = more than 23 years)	0.035	0.035	0.032	−0.030	0.095	0.3076
Gender (0 = Female; 1 = Male)	0.018	0.034	0.017	−0.043	0.077	0.5864
Modes of study (0 = Full-time; 1 = Part-time)	−0.104	0.033	−0.105	−0.170	−0.040	0.0017
Professional title (0 = Public health; 1 = Other)	−0.056	0.027	−0.073	−0.143	−0.003	0.0399
Bachelor’s Degree (0 = MUW; 1 = Other)	−0.250	0.032	−0.287	−0.359	−0.215	<0.0001
Bachelor’s studies GPA	0.387	0.030	0.447	0.380	0.515	<0.0001
Score in *Epidemiology* subtest	0.014	0.005	0.094	0.029	0.160	0.0050
Score in *Organization in Health Care* subtest	0.016	0.006	0.096	0.027	0.166	0.0067
Score in *Scientific Method* subtest	0.015	0.006	0.090	0.025	0.154	0.0063
Score in *Health and Human nutrition* subtest	0.017	0.006	0.092	0.027	0.157	0.0057
Score in *Social science* subtest	0.015	0.005	0.102	0.037	0.167	0.0022

*MUW* Medical University of Warsaw

Additional file 1 presents an assessment of whether the statistical assumptions for the above two regression models have been met.

## Discussion

In this paper, its authors have attempted to answer the question of whether the selection criteria for candidates for the Master’s studies in Public Health adopted by the MUW have the predictive value in predicting students’ future. The most important finding of the study was the fact that admissions criteria are strong predictors of academic grades (firs-year GPA and cumulative GPA). This suggests that academic success of Public Health students is closely related to the initial level of their educational potential (knowledge, skills and abilities). The results of the analyses generally conform to the findings of Lamadrid-Figueroa et al. concerning the effectiveness of candidate selection in the National Institute of Public Health of Mexico (*Instituto Nacional de Salud Pública*) [3]. With reference to the cumulative GPA, they demonstrated that the Bachelor’s studies GPA is a strong predictor of success, which confirms that it is a very effective admission criterion. As shown in this paper, the Bachelor’s studies GPA also indicated a very strong predictive ability.

The first-year GPA is very often used in predictive studies on the efficiency of admission procedure as a measure of an academic success. Recent studies show that those who perform poorly in the early years of medical school, for whatever reason, might be at an increased risk for subsequent professional misconduct [41]. The present results demonstrated that all admission criteria used at MUW had a significant impact on the academic success of students with reference to both the first-year GPA and cumulative GPA.

A comparison of both predictions of academic success presented in the present paper may show that while in the case of cumulative GPA all admission criteria were valid, their importance was lower than in the case of a success measured with the first-year GPA. Therefore, it can be assumed that results for a test entrance exam are of particular importance for a selection of students who would be at low risk of attrition after the first year due to unsatisfactory progress at school. In accordance with the list prepared by the ASPHER and concerning the necessary skills that a Public Health graduate should have, among the expected competences prevalent are those which are connected with the methodology of research and biostatistics [2, 22]. Mathematical skills are therefore a very important competence that a candidate should have to undertake Public Health studies successfully. The predictive ability of the maths subtest was demonstrated by Lamadrid-Figueroa et al. [3], whereas in this study the relevant variables were the subtests in *Epidemiology* and *Scientific Method*.

In addition to the academic variables mentioned above, some background variables (professional title and school in which Bachelor’s studies were completed) also exerted a major influence on the success measured with the first-year and cumulative GPA. This confirms theoretical assumptions of the NURS model developed by Marianne Jeffreys [32]. In this model, students’ academic success is determined by an impact of a combination of different factors, including, among others, background variables. However, not all characteristics of a student are equally important and some of them may be irrelevant in certain circumstances, such as gender or age. Based on these results, it may be assumed that, in the long-term prediction of a student’s success (cumulative GPA), the factors that are directly connected with the level of competences measured using the examination test (subtests) and the Bachelor’s studies GPA are more important than the sociodemographic variables.

The candidate’s age (age on entry) may have a potential impact on success during studies. As shown by the results of a number of studies concerning education of health professionals (e.g. nursing), the age is positively correlated with results achieved during studies [28, 42–47]. Generally, in the case of older students, significantly better results of education are noted compared with students who started studies below the age of 26, irrespectively of any additional qualifications held on entry [28, 43]. In the research on the reasons why students fail to complete their studies, young age is indicated as a negative predictor [44, 46, 47]. As reported by Pryjmachuk et al. [44] the age on entry has a moderate impact on timely graduation. Nevertheless, the results presented in this paper did not demonstrate that age had a major influence on student’s academic success.

No impact of the gender on achievements of Public Health students was noted by Lamadrid-Figueroa et al. [3]. The above observations are also confirmed by the results of this study, which may be surprising to a certain extent in light of general findings described in this respect in global literature [48–53]. As proposed by Ferguson et al. on the basis of a systematic review of literature, it is recommended to include *gender* as an important factor in prediction studies concerning medical education [54]. Most researchers show that women fare better during studies than men [53]. According to the data from US studies that are prevalent in global literature, in most standardized tests applied during entry examinations to schools of higher education, men do better than women [52, 55]. This relationship was observed neither in this paper nor in the findings reported by Lamadrid-Figueroa et al. [3]. It was not shown that the gender had any significant impact on academic success.

The assessment of the impact of the entry qualifications of a candidate (the professional title) on the likelihood of success during studies was undertaken in a number of studies related to education of health professionals [28, 43, 46]. It is difficult, however, to compare findings concerning the impact of entry qualifications from studies on education of nurses, midwives or medical rescuers with reference to recruitment for the Master’s studies in Public Health. This study shows that holding a Bachelor’s degree in Public Health seems to have a positive effect on student performance in the course of the Master’s studies. This observation is relevant as the existing admission policy rules for the Public Health program allow graduates of such diversified bachelor degree programs as Dietetics and Food Science, Electroradiology, Emergency Medicine, Nursing and Midwifery, Physiotherapy, Dental Hygiene, and Dental Technology to undertake studies. Such a broad range of entry qualifications may, however, be a hindrance to achieving education results in the course of studies that could be comparable with those achieved by graduates of the Bachelor’s studies in Public Health.

This study, contrary to expectations, does not show that holding a Bachelor’s degree in Public Health has a positive effect on results achieved during the Master’s studies. In addition, it was observed in the analysis with the first-year GPA and cumulative GPA that this entry qualification is a negative predictor. The above findings support the existing admission policy rules for the Public Health program that enable graduates of such diversified Bachelor degree programs as Dietetics and Food Science, Electroradiology, Emergency Medicine, Nursing and Midwifery, Physiotherapy, Dental Hygiene, and Dental Technology to undertake studies. The broad scope of entry qualifications does not prevent candidates from achieving, in the course of later studies, education results that are comparable with those achieved by graduates of the Bachelor’s studies in Public Health. As shown by the results of the analyses concerning the predictive value of admission criteria (total score of the MCQs exam and the Bachelor’s studies GPA), they are sufficient independent factors of a student’s academic success.

Summing up, it may be concluded that both criteria of acceptance - Bachelor’s studies GPA and entrance examination, fulfilled their role in the process of selecting the best candidates. It was evident for instance that the higher the mark average of a student in their studies of the first degree, the better were their achievements in the second degree studies. At the same time, the results of entrance examination were also of certain influence on the academic successes of the students. Despite the great evidence power of this discovery, it needs to be stressed that applying a GPA criterion during the recruitment process is not entirely flawless. The element most often pointed out to is the fact that assessment systems vary greatly at universities that offer studies of the first degree in the area of objective tools that help evaluate knowledge and skills of students. This diversity in evaluation systems in different educational institutions, particularly in the area of applying standardised evaluation method may contribute to the low level of reliability of the qualification process for candidates when this criterion is applied [56]. Bearing that fact in mind, GPA may be recommended as a criterion for acceptance, however, it ought to be complemented by another criterion (other criteria), preferably standardized ones, such as entrance examination and/or interview.

### Limitations

The selection of outcome variable may constitute one of the essential limitations of this predictive analysis. Since academic success may be measured in a variety of ways, first-year GPA and cumulative GPA do not constitute the only measures of success. Therefore, other variables describing academic success, including those referring to events occurring after graduation, also need to be considered.

In addition, data for this study have been sourced from one medical university with all limitations this entails, including the difficulty with assessing whether the adopted criteria could be equally valid in similar degree programs at other universities. Therefore, to ensure reliability, other academic institutions should determine their own predictors of academic success with regard for their own specific pools of students and available measures. However, due to the lack of available results of this type of research relating to education of Public Health professionals, it seems important to present to the members of the European academic environment the issues related to the correct evaluation of tools used to select candidates for the Public Health program. Harmonisation with respect to key competences [2] and standardisation of education as part of the advancing implementation of the provisions of the Bologna Declaration allow one to argue that even experiences of individual academic centres may be a valuable source of information for other participants of the European Higher Education Area (EHEA).

## Conclusions

The admission criteria of the total score of the MCQs exam and the Bachelor’s studies GPA may be successfully applied while selecting candidates for the Master’s studies in Public Health. The high predictive validity of the applied admission system confirms that the admission policy adopted at the MUW is accurate. It is still necessary, however, to develop a well-planned recruitment strategy that would meet new needs connected with the growing number of competences that are required of Public Health professionals.

## Additional file

Additional file 1: Testing the assumptions of linear regression. (DOCX 95 kb)
