# Supplementary material for: Evidence-based selection process to the Master of Public Health program at Medical University
Source: BMC Med Educ. 2017 Sep 11;17:157. doi: 10.1186/s12909-017-1007-z (PMC5594576; doi:10.1186/s12909-017-1007-z)
Supplement: Additional file 1: — Testing the assumptions of linear regression. (DOCX 95 kb) [file 12909_2017_1007_MOESM1_ESM.docx]

**Testing the assumptions of linear regression**

Quantitative predictors (age, the bachelor’s studies GPA, the score result of the entry examination broken down by five theme areas) demonstrated a negative skewness of the distribution. Single cases with low values (skewness between -0.180 and -0.038) were found. These variables were transformed (the Box–Cox transformation) to obtain a distribution close to a normal distribution that was appropriate for a development of linear regression models. In addition, no correlation (VIF < 10, Table 1) between the independent variables (background and academic variables) was found. Good adjustment of independent variables to the regression model was assessed with the use of the Ramsey RESET test (Regression Equation Specification Error Test). The form of regression models I and II was correct (F = 0.3343, P = 0.7159 and F = 0.2895, P = 0.7487 for model I and model II, respectively), which showed good adjustment of variables to linear models. The models were later assessed with reference to the presence of outliers (a poor candidate with a high GPA). The Mahalanobis and Cook’s distances did not demonstrate a significant impact of outliers on the quality of prediction of the first-year GPA or cumulative GPA (outcome variables).

Since the quality of the prediction of the first-year GPA or cumulative GPA strongly depends on residuals (differences between the observed and predicted values of GPA), a detailed analysis of residuals for both models was conducted. A uniform degree of variability of results (dispersion of residuals, variance) for low and high values of the first-year GPA or cumulative GPA was confirmed with the White test (Table 3). In addition, it was confirmed that the residuals for both models had their distributions close to the normal distribution (Jarque-Bera test, Table 2 and Fig. 1). The lack of autocorrelation was the last criterion met in the analysis of residuals (the Ljung-Box test, Table 3). All of the above gave the grounds for predictive analysis of the academic success using the linear regression model and estimating regression coefficients with the OLS model.

**Table S1.** Assessment of the degree of the predictor correlation in the multicollinearity test.

| Variable | Variance inflation factor |
| --- | --- |
| Age on entry | 1.126 |
| Gender | 1.041 |
| Modes of study | 1.221 |
| Professional title | 1.405 |
| Bachelor’s Degree | 1.489 |
| Bachelor’s studies GPA | 1.322 |
| Score in *Epidemiology* subtest | 1.248 |
| Score in *Organization in Health Care* subtest | 1.397 |
| Score in *Scientific Method* subtest | 1.190 |
| Score in *Health and Human nutrition* subtest | 1.225 |
| Score in *Social science* subtest | 1.225 |

**Table S2.** Results of the residuals analysis for the two tested linear regression models.

| Analysis of residuals | Regression model with the dependent variable: | |
| --- | --- | --- |
|  | **First-year GPA** | **Cumulative GPA** |
| Homoscedasticity  (White test) | W = 19.9853  P = 0.4588 | W = 24.9205  P = 0.2045 |
| Normal distribution of residuals  (Jarque-Bera test) | JB = 4.1445  P = 0.1261 | JB = 0.5663  P = 0.7534 |

**Table S3.** Autocorrelation function for 15 delays with data sorted according to timestamps assigned to dates of applications of student candidates.

| Delay | Regression model with the dependent variable: | | | | | |
| --- | --- | --- | --- | --- | --- | --- |
|  | **First year GPA** | | | **Cumulative GPA** | | |
|  | **AC** | **Ljung*-*Box Q** | ***P*-value** | **AC** | **Ljung*-*Box Q** | ***P*-value** |
| 1 | -0.057 | 1.939 | 0.164 | -0.002 | 0.003 | 0.954 |
| 2 | 0.030 | 2.485 | 0.289 | 0.013 | 0.110 | 0.946 |
| 3 | -0.009 | 2.531 | 0.470 | -0.015 | 0.240 | 0.971 |
| 4 | 0.028 | 2.998 | 0.558 | 0.020 | 0.487 | 0.975 |
| 5 | 0.001 | 2.999 | 0.700 | 0.027 | 0.906 | 0.970 |
| 6 | 0.047 | 4.319 | 0.634 | 0.017 | 1.076 | 0.983 |
| 7 | 0.047 | 5.659 | 0.580 | 0.035 | 1.785 | 0.971 |
| 8 | -0.046 | 6.945 | 0.543 | -0.045 | 2.978 | 0.936 |
| 9 | 0.073 | 10.097 | 0.343 | 0.065 | 5.477 | 0.791 |
| 10 | 0.014 | 10.208 | 0.422 | 0.012 | 5.564 | 0.850 |
| 11 | -0.005 | 10.222 | 0.511 | -0.021 | 5.837 | 0.884 |
| 12 | -0.013 | 10.325 | 0.587 | -0.001 | 5.838 | 0.924 |
| 13 | -0.011 | 10.396 | 0.661 | 0.037 | 6.649 | 0.919 |
| 14 | 0.021 | 10.657 | 0.713 | 0.012 | 6.733 | 0.944 |
| 15 | 0.008 | 10.699 | 0.774 | 0.045 | 7.955 | 0.926 |

AC - autocorrelation

**
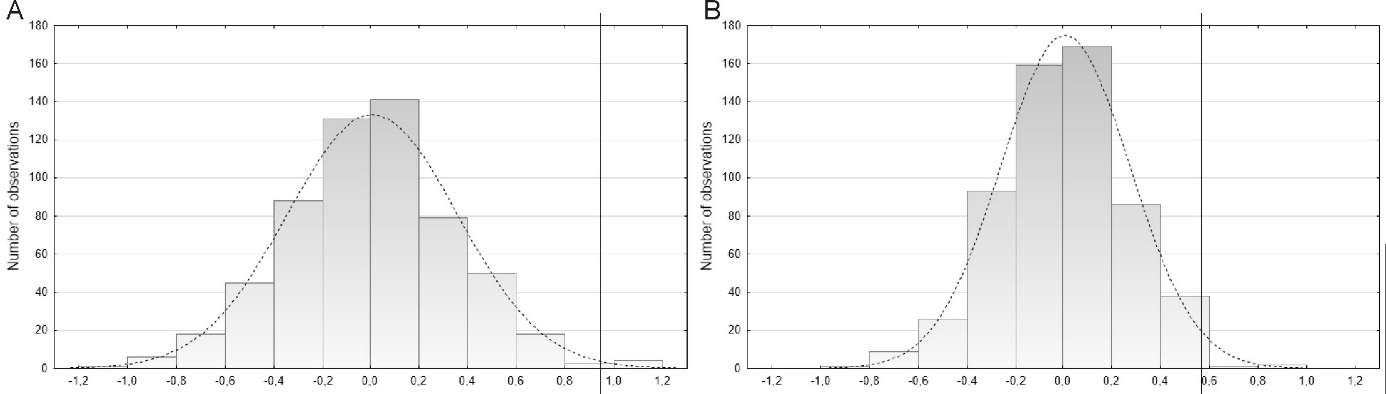
**

**Figure S1.** Distribution of standardized residuals for the regression model with the dependent variable: (A) First-year GPA; (B) Cumulative GPA
